# Machine Learning-Based Approaches for Prediction of Patients’ Functional Outcome and Mortality after Spontaneous Intracerebral Hemorrhage

**DOI:** 10.3390/jpm12010112

**Published:** 2022-01-14

**Authors:** Rui Guo, Renjie Zhang, Ran Liu, Yi Liu, Hao Li, Lu Ma, Min He, Chao You, Rui Tian

**Affiliations:** 1 Department of Neurosurgery, West China Hospital, Sichuan University, Chengdu 610041, China; 13980774725@163.com (R.G.); zrjwch@163.com (R.Z.); liuyi@wchscu.cn (Y.L.); coscolh@126.com (H.L.); alex80350305@163.com (L.M.); heminhx@aliyun.com (M.H.); youchao@vip.126.com (C.Y.); 2 Department of Clinical Medicine, West China Medical College, Sichuan University, Chengdu 610041, China; 3 Engineering Research Center of Medical Information Technology, Ministry of Education, West China Hospital, Sichuan University, Chengdu 610041, China; 283897787@163.com; 4 West China Brain Research Centre, West China Hospital, Sichuan University, Chengdu 610041, China

**Keywords:** spontaneous intracerebral hemorrhage (SICH), machine learning, 90-day function outcome, mortality

## Abstract

Spontaneous intracerebral hemorrhage (SICH) has been common in China with high morbidity and mortality rates. This study aims to develop a machine learning (ML)-based predictive model for the 90-day evaluation after SICH. We retrospectively reviewed 751 patients with SICH diagnosis and analyzed clinical, radiographic, and laboratory data. A modified Rankin scale (mRS) of 0–2 was defined as a favorable functional outcome, while an mRS of 3–6 was defined as an unfavorable functional outcome. We evaluated 90-day functional outcome and mortality to develop six ML-based predictive models and compared their efficacy with a traditional risk stratification scale, the intracerebral hemorrhage (ICH) score. The predictive performance was evaluated by the areas under the receiver operating characteristic curves (AUC). A total of 553 patients (73.6%) reached the functional outcome at the 3rd month, with the 90-day mortality rate of 10.2%. Logistic regression (LR) and logistic regression CV (LRCV) showed the best predictive performance for functional outcome (AUC = 0.890 and 0.887, respectively), and category boosting presented the best predictive performance for the mortality (AUC = 0.841). Therefore, ML might be of potential assistance in the prediction of the prognosis of SICH.

## 1. Introduction

Spontaneous intracerebral hemorrhage (SICH), which accounts for 10–30% of all strokes, is the most fatal and disabling type of hemorrhage [1,2,3]. China has one of the highest disease burdens of SICH in the world [1,4]. Because of the high disability and mortality rates of SICH, outcome-prediction models combining clinical presentations, laboratory data and imaging findings are of great significance and can ensure the optimal care [5]. Several prognostic tools have been proposed for outcome prediction in intracerebral hemorrhage (ICH) such as ICH score [6]. These tools are potentially useful for predicting prognosis, facilitating communication between clinicians, and selecting patients for interventions [7,8,9]. However, the predictive performance of the 90-day functional outcome and mortality of these tools remains unknown. Besides, the ICH score only consists of the Glasgow Coma Scale (GCS), ICH volume, age, location, and intraventricular extension of the hematoma [6]. Recent studies showed that some laboratory results, such as levels of monocytes and lymphocytes [10,11,12,13,14], offered potential predictive benefits to the outcome of SICH, suggesting that a more accurate model could be made including more variables. Moreover, there is still no widely recognized tool for predicting the prognosis of Chinese SICH patients [15].

As a type of artificial intelligence, machine learning (ML) has several advantages in detecting the possible interactions among attributes and may be useful in the identification of prognostic markers. The key feature of ML is to allow computers to detect underlying patterns by iteratively learning from data, based on which a new model can be created, which prevents the influence from the researchers’ intervention. In recent years, ML have been widely applied to the outcome prediction models for cerebrovascular diseases such as ischemic stroke [16,17], aneurysmal subarachnoid hemorrhage [18], and arteriovenous malformations [19]. However, ML-based outcome-prediction models for the SICH in Chinese patients are still rare. The aim of this study was to develop a prognostic model with ML methods to predict the functional outcome and mortality in Chinese patients with SICH according to the initial information on admission to hospital and to compare them with ICH score, the traditional risk stratification scale.

## 2. Materials and Methods

### 2.1. Study Population

We retrospectively reviewed SICH patients admitted to West China Hospital during a 2-year period, from 1 January 2018, to 31 December 2019. The diagnosis of SICH was confirmed by head computed tomography (CT) within the first 24 h after admission.

All continuous patients who were diagnosed with SICH during this period and were followed up for more than 3 months were included for further analysis. Extremely severe cases whose families refused any therapy after diagnosis were excluded in this study.

### 2.2. Data Collection

The study was conducted according to the guidelines of the Declaration of Helsinki and was approved by the Ethics Committee of West China Hospital (protocol code 1.1; 1 July 2017). The data used to develop the ML models were collected from the electronic medical records, including clinical, radiographic, and laboratory variables at the first evaluation. The demographic information, vital signs, radiographic findings, laboratory results, previous medical history, and treatments were collected. The first vital signs (body temperature [BT], heart rate [HR], and blood pressure) after hospital arrival were used. Length of time in the emergency room (ER) meant the period from when the patient first arrived ER to when the patients were transferred to the neurosurgery department or the operating room. The level of consciousness was assessed with GCS. Location of the hematoma (supratentorial, infratentorial, and both supra- and infratentorial), intraventricular hemorrhage (IVH), and the initial hematoma volume were evaluated by CT scan independently by two experienced doctors. The hematoma volume was measured using the ABC/2 method [20], in which A is the greatest diameter on the largest hemorrhage slice, B is the diameter perpendicular to A, and C is the approximate number of axial slices with hemorrhage multiplied by the slice thickness. Levels of complete blood count, blood glucose (BG), triglyceride, total cholesterol, high density lipoprotein cholesterol, low density lipoprotein cholesterol, creatinine, uric acid, sodium, chlorine, fibrinogen, and D-dimer were evaluated in the laboratory of our hospital. Estimated glomerular filtration rate (eGFR) was calculated based on the Chronic Kidney Disease Epidemiology Collaboration (CKD-EPI) equation. The previous medical history, including hypertension, diabetes mellitus (DM), coronary heart disease, kidney diseases, and pulmonary diseases, was obtained by the patients’ self-reports or the medical treatment they received.

### 2.3. In-Hospital Treatments and Outcomes

In-hospital treatments included conservative treatment or surgery (surgical hematoma evacuation). Generally, patients who had a supratentorial hematoma of ≥30 mL or infratentorial hematoma of ≥10 mL were recommended for surgery.

All patients were followed up for at least 3 months. The primary outcome was the functional disability at the 3rd month evaluated by the modified Rankin Scale ([mRS] from 0, no functional deficit, to 6, death). An mRS of 0–2 was defined as a favorable functional outcome, while an mRS of 3–6 was defined as an unfavorable functional outcome in this study. Survival at the 3rd month was evaluated as the secondary outcome.

### 2.4. Machine Learning ML Algorithms

Firstly, all candidate variables were tested with univariate analysis.

Subsequently, recursive feature elimination with cross-validation (RFECV) was used to obtain the best feature combination for each model. RFECV included two parts: recursive features elimination (RFE) and cross-validation. Given an external estimator, RFE was used to select features by recursively considering increasingly small sets of features. For each ML algorithm, firstly, the estimator was trained on the initial set of features which contained all 41 variables, and the importance of each feature was obtained. Then, the least important feature was pruned from the current set of features. This procedure was recursively repeated on the pruned set until the optimal combination of features was got.

Six ML algorithms, which are efficient and widely used methods for the binary classification, were used in this study. Logistic regression (LR) and LRCV are most wildly used statistical models which in their basic form use a logistic function to model a binary dependent variable [21]. LR and LRCV are of high efficiency, especially for analogously linear datasets, and they are much faster in training models than other ML-based algorithms like support vector machine (SVM) and random forest (RF). SVM is one of the most robust prediction methods, being based on statistical learning frameworks or the Vapnik–Chervonenkis theory. It can efficiently perform not only a linear classification but also a non-linear classification using the kernel trick [22]. RF operates by constructing a multitude of decision trees at training time. For classification tasks, the output of the RF is the class selected by most trees [23]. RF is usually flexible and easy to use in various conditions. Extreme gradient boosting (XGBoost) and category boosting (CatBoost) are typical and widely used ensemble learning algorithms. Ensemble methods use multiple learning algorithms to obtain a better predictive performance than that which could be obtained from any of the constituent learning algorithms alone [24].

In the current study, a five-fold cross-validation was used to build and assess the LR, LRCV, SVM, RF, XGBoost, and CatBoost models. All samples were divided into five approximately equally sized subsamples. Four subsamples were used as training data and the remaining one subsample was retained as the validation set for testing the models. The process was then repeated five times, with each of the five sub-samples used exactly once for validation. The five results from the repetition were then averaged to produce a final estimation. The area under the receiver operator characteristic curve (AUC) was used to evaluate the predictive performance of each model.

### 2.5. Comparison to the Intracerebral Hemorrhage (ICH) Score

The ICH score was calculated as described previously [6] based on GCS, ICH volume, IVH, location of the hematoma, and age. Its performance (AUC) was compared with the developed ML-based models using a pairwise *t*-test which was commonly used in the previous studies to assess the performance [25,26,27].

### 2.6. Statistical Analysis

All statistical analyses were performed in Python programming language, version 3.7 (Python Software Foundation). Qualitative data are described as the frequency and percentage. Fisher’s exact test or Chi-square test were used to compare the categorical variables in subgroups. Quantitative data were first tested for normality by the D’Agostino–Pearson test. Normal data are expressed as the mean ± standard deviation (SD), while non-normal data are displayed as the median and interquartile range (IQR). Student’s *t*-test was used for the comparison of normal variables, while the Wilcoxon test was used for the comparison of non-normal variables. The performance (AUC) of the different models was compared using the pairwise *t*-test. For all the statistical hypothesis, *p* values < 0.05 were considered significant.

## 3. Results

### 3.1. Patient Characteristics

**Figure 1:**
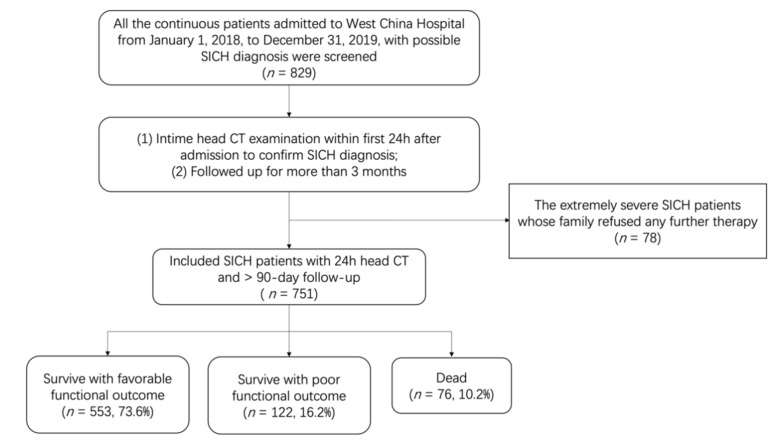
Flowchart of SICH patient inclusion and exclusion.

As shown in Figure 1, a total of 829 patients admitted with the diagnosis of SICH in our hospital during the 2-year period (from 1 January 2018, to 31 December 2019) were retrospectively reviewed. Seventy-eight patients were excluded because their family refused any further therapy after the diagnosis. The remaining 751 patients were further analyzed. The overall 90-day mortality was 10.2% (*n* = 76), while 553 patients (73.6%) presented favorable functional outcome at 90-day follow up. The cohort characteristics were presented in Table 1. The raw data supporting the conclusions of this article will be made available by the authors through contacting the corresponding author, without undue reservation.

### 3.2. Predictive Performance of the ML-Based Models

The intact algorithms for all the models with the optimal parameters were shown in the Supplementary Materials.

**Figure 2:**
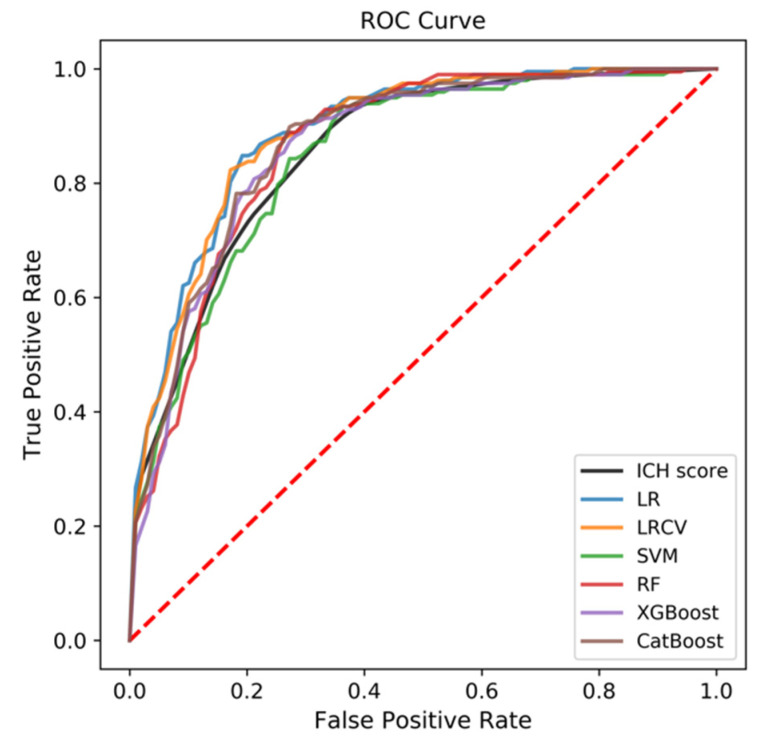
The receiver operating characteristic (ROC) curve of all the six machine learning (ML)-based models compared with the traditional ICH Score, with respect to predictive performance for the functional outcome at the third month.

Among all the ML-based models, LR and LRCV showed the best predictive performance for the functional outcome at the 3rd month (AUC = 0.890 and 0.887, respectively, Table 2 and Figure 2), followed by CatBoost, XGBoost, RF, and SVM (AUC = 0.871, 0.864, 0.862, 0.849, respectively). In both LR and LRCV models, location of the hematoma, coagulation disorders, AMC, GCS, and intraventricular hemorrhage contributed materially to the models (Table 3).

**Figure 3:**
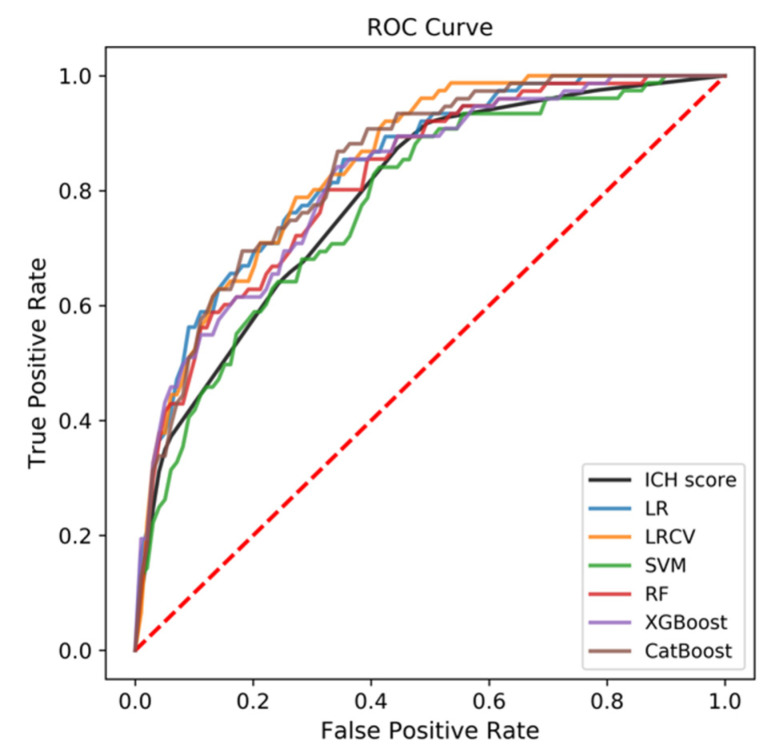
The ROC curve of all the six ML-based models compared with the traditional ICH Score, with respect to predictive performance for the 90-day mortality at the third month.

The predictive performance for the 90-day mortality was assessed by the similar method. As shown in Table 2 and Figure 3, CatBoost and LRCV provided the best predictive performance for the mortality outcome (AUC = 0.841 and 0.844, respectively). The AUCs of the other four models were as follows: LR, 0.837; XGBoost, 0.820; RF, 0.818; SVM, 0.777. As shown in Table 3, GCS, Age, D-dimer, and HR contributed largely to CatBoost, while AMC, location of the hematoma, and history of diabetes mellitus contributed significantly to LRCV.

### 3.3. Comparison to ICH Score

As shown in Supplementary Table S1, predictive performance for the functional outcome of LR (AUC = 0.890, *p* < 0.001) and LRCV (AUC = 0.887, *p* = 0.001) were significantly better than that of ICH score (AUC = 0.856). Besides, CatBoost (AUC = 0.841, *p* = 0.03) and XGBoost (AUC = 0.820, *p* = 0.05) showed significantly better performance to predict the 90-day mortality than ICH score (AUC = 0.790).

## 4. Discussion

The prognosis prediction of SICH has long been dependent on the ICH score. Recent studies revealed the promising role of some laboratory results (such as levels of monocytes and lymphocytes) in the SICH outcome prediction. However, the ICH score, a traditional and widely-used prognostic predictive method, consists of the GCS, ICH volume, age, location, and intraventricular extension of the hematoma [6], without involvement of any laboratory results. In this study, we built distinctive ML-based models to develop a more accurate model involving multiple variables, in order to predict the 90-day functional outcome and mortality with better efficacy.

In this study, we developed 6 ML-based models for predicting the outcome of SICH. We analyzed the clinical characteristics, radiographic results, laboratory results, and previous medical history of 751 consecutive SICH patients by reviewing their medical records. The results showed that LR and LRCV were the most accurate models to predict the functional outcome with an AUC of 0.890 and 0.887, respectively, both of which were significantly better than that of ICH score. Besides, CatBoost and LRCV showed the best performance in the prediction of the 90-day mortality (AUC = 0.841 and 0.844, respectively), and they were also significantly more accurate than ICH score.

Patients with the favorable functional outcome were significantly different from those with the unfavorable functional outcome in 15 variables, including age (younger), location of the hematoma (supratentorial), initial hematoma volume (smaller), IVH (without), GCS (greater), BT (lower), HR (lower), BG (lower), creatinine (lower), chlorine (higher), eGFR (higher), WBC (lower), absolute neutrophil count (ANC, lower), absolute monocyte count (AMC, lower), and D-dimer (lower, *p* = 0.004, <0.001, <0.001, <0.001, <0.001, <0.001, 0.001, <0.001, 0.004, 0.002, <0.001, <0.001, <0.001, 0.006, <0.001, respectively).

Similar results were shown when considering 90-day mortality (Table 1). Patients who survived 90 days after SICH were significantly different from the others in age (younger), location of the hematoma (supratentorial), hematoma volume (smaller), GCS (higher), time in ER (shorter), BT (lower), HR (lower), DM (without), BG (lower), creatine (lower), uric acid (lower), triglyceride (lower), chlorine (higher), eGFR (higher), WBC (lower), ANC (lower), AMC (lower), and D-dimer (lower).

Both the univariate analysis and the feature importance analysis of the ML-based models illuminated that the level of the absolute monocyte cells provided a significant contribution to the prediction of both 90-day mortality and functional outcome. Higher levels of monocytes indicated a poor outcome of SICH. The recruitment of monocytes is a key feature of inflammation [28]. In 2016, Morotti et al. [10] illuminated that a higher level of monocyte on admission was directly associated with a higher risk of hematoma expansion, which might suggest a more unfavorable outcome. Indeed, many previous studies concluded that an elevated level of the monocyte was an independent risk factor for 30-day mortality in SICH patients, suggesting that monocyte level on admission might help predict the outcome of SICH [11,12,13,14], which was consistent with our study. Using ML technology, the monocyte level was proved to have significant predictive benefit of the 90-day outcome of SICH, which also suggested that additional knowledge could be obtained, benefiting from ML algorithms.

In clinical practice, the most widely used risk stratification scale for ICH, the ICH score, consists of GCS, ICH volume, age, location, and intraventricular extension of the hematoma [6]. The ICH score predicts the 30-day mortality after ICH. As our results displayed, some ML-based models performed significantly better than the ICH score in predicting both 90-day functional outcome and 90-day mortality. Overall, our results demonstrate how the data mining approach can be used as an alternative to the conventional approach, achieving comparable performance to well accepted prognostic models.

In this study, RFE was used to select the optimal combination of features by recursively considering smaller and smaller sets of features according to the importance, which enumerated almost all the combinations. Although this method is not so efficient, it is the best way to improve the performance of the model. Besides RFE, minimum redundancy maximum relevance (MrMr) and the Boruta algorithm are also efficient and widely-used methods for feature selection. According to Peng et al., MrMr can use either mutual information, correlation, or distance/similarity scores to select features. However, this algorithm may underestimate the importance of each of the seemingly insignificant variables with poor performance, which may turn significant when organized into ML-based models. Thus, MrMr is mostly used when variables are categorical. However, there are many quantitative variables in our datasets. Similar to MrMr, the Boruta algorithm optimizes the combination of variables by reducing the relevancy between the selected variables and increasing the relevancy between the variables and outcomes. Although these methods are more efficient in feature selection, RFE can provide a better performing model by enumeration.

This study has several clinical and methodological implications. Firstly, the factors which were previously neglected could be discovered. Together with these factors, the predictive performance could be improved using machine learning approaches. Secondly, the best model in the present study only contained a small number of variables. Thus, these models can be used easily in clinical practice to provide an accessible prediction of the outcome in SICH patients, which helps both the doctors and patients’ families to choose the optimal management. Based on our studies, online websites were developed [http://114.251.235.51:1226/ich_recover_predict (accessed on 2 January 2022) for 90-day functional outcome; http://114.251.235.51:1226/ich_death_predict (accessed on 2 January 2022) for 90-day mortality]. Furthermore, our results eliminated that the predictive performance of the ML-based models remained high even when plenty of variables were input. Nowadays, since the electronic medical records are widely used, much larger datasets are needed to be manipulated in the future. ML algorithms are much more suitable to deal with the increasing number of variables than the traditional statistical methods.

However, our study had several limitations. First, some patients in critical conditions were not included in the study because of early withdrawal of care. Second, the sample size of our retrospective study may limit the improvement of the model performance. Third, the primary aim of this study was to predict the 90-day outcome of ICH patients based on the initial information on admission to hospital, thus serial changes of variables after admission were not considered. Moreover, external validation is lacking in the present study, which may restrict the generalizability of our results. Future studies with larger samples may help provide a higher predictive power.

## 5. Conclusions

In conclusion, the prediction of functional outcome and mortality after SICH is a challenge. Our findings suggested that the ML-based model is of high potential. The CatBoost and LRCV models are of good predictive performance for 90-day mortality with considerable accuracy, while the LRCV and LR models are of reliable predictive performance for 90-day functional outcome, all of which were better than ICH score, the traditional and widely-used risk stratification scale. These models might provide additional assistance in the prediction of functional outcome or mortality for SICH patients.

## Figures and Tables

**Table 1:** Clinical characteristics of the patients with spontaneous intracerebral hemorrhage (SICH).

Variables	Functional Outcome			Mortality		
	Favorable (*n* = 553)	Unfavorable (*n* = 198)	*p*-Value	Survival (*n* = 675)	Death (*n* = 76)	*p*-Value
Demographics						
Age, years	54.0 (46.0–66.0)	58.9 (43.7–74.0)	0.004 **	54.0 (46.0–66.0)	65.5 (52.5–77.0)	<0.001 ***
Gender, *n* (%)			0.70			0.20
Female	189 (74.70%)	64 (25.30%)		232 (92.06%)	20 (7.94%)	
Male	364 (73.09%)	134 (26.91%)		443 (88.78%)	56 (11.22%)	
Clinical features						
Location, *n* (%)			<0.001 ***			<0.001 ***
Supratentorial	475 (78.51%)	130 (21.49%)		556 (91.90%)	49 (8.10%)	
Infratentorial	72 (58.06%)	52 (41.94%)		106 (85.48%)	18 (14.52%)	
Supra and Infra	6 (27.27%)	16 (72.73%)		13 (59.09%)	9 (40.91%)	
Initial volume, mL	25.0 (15.0–35.0)	34.9 (19.3–50.5)	<0.001 ***	25.0 (15.0–35.0)	35.0 (20.0–46.2)	<0.001 ***
IVH, *n* (%)			<0.001 ***			0.001 **
Yes	253 (63.73%)	144 (36.27%)		342 (86.15%)	55 (13.85%)	
No	300 (84.75%)	54 (15.25%)		333 (94.07%)	21 (5.93%)	
GCS	13 (9–15)	8 (6–8)	<0.001 ***	13 (8–15)	7 (4–10)	<0.001 ***
Length of time in ER, h	1.08 (0.57–2.35)	1.13 (0.65–2.35)	0.48	1.03 (0.57–2.35)	1.47 (0.85–2.35)	0.02 *
BT, °C	36.6 (36.5–36.8)	36.8 (36.5–37.0)	<0.001 ***	36.6 (36.5–36.9)	36.8 (36.5–37.0)	0.02 *
HR, bpm	82 (72–92)	86 (75 -102)	0.001 **	82 (72–93)	94 (80–112)	<0.001 ***
Systolic BP, mmHg	165 (144–183)	164 (130–199)	0.48	165 (144–182)	168 (128 -208)	0.18
Diastolic BP, mmHg	96 (82–107)	92 (81–109)	0.10	96 (82–108)	93.5 (78–107)	0.12
Medical history						
Hypertension, *n* (%)			0.48			0.24
Yes	429 (72.96%)	159 (27.04%)		524 (77.63%)	64 (82.89%)	
No	124 (76.07%)	39 (23.93%)		151 (22.37%)	12 (15.79%)	
DM, *n* (%)			0.09			0.007 **
Yes	50 (64.94%)	27 (35.06%)		62 (80.52%)	15 (19.48%)	
No	503 (74.63%)	171 (25.37%)		613 (90.95%)	61 (9.05%)	
Coronary heart disease, *n* (%)			0.21			0.29
Yes	34 (65.38%)	18 (34.62%)		44 (84.62%)	8 (15.38%)	
No	519 (74.25%)	180 (25.75%)		631 (90.27%)	68 (9.73%)	
Kidney diseases, *n* (%)			0.16			0.15
Yes	30 (63.83%)	167 (36.17%)		38 (82.61%)	8 (17.39%)	
No	523 (74.29%)	181 (25.71%)		637 (90.35%)	68 (9.65%)	
Pulmonary diseases, *n* (%)			0.07			0.15
Yes	68 (66.02%)	35 (33.98%)		88 (85.44%)	15 (14.56%)	
No	485 (74.85%)	163 (25.15%)		587 (90.59%)	61 (9.41%)	
Cigarette smoking, *n* (%)			0.43			0.31
Yes	175 (75.76%)	56 (24.24%)		212 (91.77%)	19 (8.23%)	
No	378 (75.76%)	142 (27.31%)		463 (89.04%)	57 (10.96%)	
Alcohol consumption, *n* (%)			0.41			0.76
Yes	170 (75.89%)	54 (24.11%)		203 (90.62%)	21 (9.38%)	
No	383 (72.68%)	144 (27.32%)		472 (89.56%)	54 (10.44%)	
Family history of stroke, *n* (%)			0.19			0.74
Yes	11 (57.89%)	8 (42.11%)		18 (94.74%)	1 (5.26%)	
No	542 (74.04%)	190 (25.96%)		657 (89.75%)	75 (10.25%)	
Coagulative disorders, *n* (%)			0.05			0.86
Yes	6 (46.15%)	7 (53.85%)		11 (84.62%)	2 (15.38%)	
No	547 (74.12%)	191 (25.88%)		664 (89.97%)	74 (10.03%)	
Anticoagulation therapy, *n* (%)			0.19			0.66
Yes	11 (57.89%)	8 (42.11%)		16 (84.21%)	3 (15.79%)	
No	542 (74.04%)	190 (25.96%)		659 (90.03%)	73 (9.97%)	
Antiplatelet therapy, *n* (%)			0.61			0.07
Yes	2 (50.00%)	2 (50.00%)		2 (50.00%)	2 (50.00%)	
No	551 (73.76%)	196 (26.24%)		673 (90.09%)	74 (9.91%)	
Laboratory studies						
BG, mmol/L	7.16 (6.07–8.85)	9.25 (7.35–11.64)	<0.001 ***	7.38 (6.24–9.41)	9.37 (7.35–12.45)	<0.001 ***
Creatinine, µmol/L	69 (56–84)	72 (60–96)	0.004 **	69 (56–85)	79 (64–116)	<0.001 ***
Uric acid, µmol/L	324 (250–407)	338 (257–419)	0.26	321 (250–407)	348 (288–439)	0.03 *
TG, mmol/L	1.14 (0.80–1.72)	1.21 (0.87–1.73)	0.09	1.13 (0.81–1.69)	1.38 (0.88–1.99)	0.03 *
Cholesterol, mmol/L	4.42 (3.78–5.06)	4.34 (3.68–5.06)	0.36	4.40 (3.76–5.06)	4.36 (3.66–5.12)	0.50
HDLC, mmol/L	1.29 (1.03–1.61)	1.33 (1.03–1.66)	0.19	1.30 (1.04–1.63)	1.31 (1.02–1.61)	0.33
LDLC, mmol/L	2.60 (2.08–3.21)	2.51 (1.91–3.24)	0.11	2.60 (2.05–3.21)	2.40 (1.83–3.3)	0.07
Sodium, mmol/L	138.4 (136.1–140.3)	138.3 (134.0–142.6)	0.29	138.4 (136.1–140.4)	137.9 (133.6–142.3)	0.45
Chlorine, mmol/L	101.4 (98.8–104.3)	100.5 (95.5–105.4)	0.002 **	101.3 (98.6–104.3)	99.6 (94.8–104.4)	0.001 **
eGFR, mL/min	91.0 (87.7–103.5)	91.0 (77.2–100.9)	<0.001 ***	91.0 (87.0–103.6)	86.0 (63.6–91.0)	<0.001 ***
Platelet, 10^9^ cells/L	170 (129–217)	184 (136–222)	0.12	175 (131–218)	175 (98–252)	0.35
WBC, 10^9^ cells/L	10.11 (7.58–12.99)	11.91 (9.22–15.43)	<0.001 ***	10.54 (7.76–13.22)	11.62 (8.24–16.45)	0.006 **
ANC, 10^9^ cells/L	8.43 (5.67–11.25)	10.33 (7.09–13.26)	<0.001 ***	8.81 (5.91–11.51)	9.64 (6.17–13.57)	0.03 *
ALC, 10^9^ cells/L	1.09 (0.76–1.48)	1.17 (0.72–1.82)	0.08	1.09 (0.75–1.51)	1.19 (0.73–1.99)	0.06
AMC, 10^9^ cells/L	0.39 (0.26–0.53)	0.42 (0.28–0.62)	0.006 **	0.4 (0.26–0.54)	0.47 (0.30–0.62)	0.02 *
Hematocrit	0.41 (0.38–0.44)	0.41 (0.37–0.44)	0.29	0.41 (0.38–0.44)	0.42 (0.37–0.44)	0.23
Fibrinogen, g/L	2.77 (2.26–3.41)	2.74 (2.16–3.57)	0.44	2.75 (2.24–3.42)	2.77 (2.28–3.61)	0.27
D-dimer, mg/L FEU	0.64 (0.31–1.94)	1.43 (0.63–2.84)	<0.001 ***	0.72 (0.32–2.16)	2.37 (0.80–5.24)	<0.001 ***
Treatment, *n* (%)			0.92			0.74
Surgery	172 (73.19%)	63 (26.81%)		213 (90.64%)	22 (9.36%)	
Conservative	381 (73.84%)	135 (26.16%)		462 (89.53%)	54 (10.47%)	

* *p* < 0.05; ** *p* < 0.01; *** *p* < 0.001. ANC, absolute neutrophil count; ALC, absolute lymphocyte count; AMC, absolute monocyte count; BG, blood glucose; BT, body temperature; DM, diabetes mellitus; eGFR, estimated glomerular filtration rate; ER, emergency room; GCS, Glasgow Coma Scale; HR, heart rate; IVH, intraventricular hemorrhage; TG, triglyceride; WBC, white blood cell; HDLC, high-density lipoprotein cholesterol; LDLC, low-density lipoprotein cholesterol.

**Table 2:** Predictive performance for the 90-day functional outcome and mortality after spontaneous intracerebral hemorrhage.

Algorithm	Functional Outcome		Mortality	
	AUC, Mean	AUC, 95%CI	AUC, Mean	AUC, 95% CI
ICH score	0.856	0.827–0.884	0.790	0.712–0.867
LR	0.890	0.858–0.922	0.837	0.780–0.894
LRCV	0.887	0.855–0.920	0.844	0.807–0.881
SVM	0.849	0.804–0.894	0.777	0.720–0.833
RF	0.862	0.813–0.912	0.818	0.718–0.917
XGBoost	0.863	0.815–0.911	0.820	0.741–0.899
CatBoost	0.871	0.829–0.913	0.841	0.774–0.907

AUC, area under the receiver operator characteristic curve; CatBoost, Category Boosting; CI, confidence interval; ICH, intracerebral hemorrhage; LR, logistic regression; SD, standard deviation; SVM; support vector machine; RF, random forest; XGBoost, extreme gradient boosting.

**Table 3:** List of variables used in the final model.

Algorithm	Variables for Functional Outcome ^a^	Variables for Mortality ^a^
LR	Coagulation disorders, Location of the hematoma, GCS, IVH, AMC, BG, BT, D-dimer, Age, ANC, Chlorine	Location of the hematoma, AMC, GCS, DM, WBC, D-Dimer, ANC, BG, Age, Chlorine, IVH, HR, Time in ER, BT
LRCV	Coagulation disorders, Location of the hematoma, AMC, GCS, IVH, BG, ANC, WBC, D-dimer, Age, BT	AMC, Location of the hematoma, DM, GCS, WBC, ANC, IVH, D-Dimer, Age, Chlorine, BG, TG, HR, Hematoma volume, BT
SVM ^b^	-	-
RF	GCS, BG, Hematoma volume, Location of the hematoma, D-Dimer, IVH	GCS, D-dimer, Age, BG, HR, eGFR, Time in ER, Hematoma volume, Chlorine, ANC, WBC, Location of the hematoma, Creatine, Uric acid, TG, BT, IVH, DM
XGBoost	GCS, BG, D-dimer, Location of the hematoma, eGFR, Hematoma volume, Age, WBC, Creatine, Chlorine	GCS, D-dimer, Age, WBC, Location of the hematoma, Hematoma volume, eGFR, HR, Chlorine, Time in ER, Creatine, ANC, TG
CatBoost	GCS, BG, D-dimer	GCS, Age, D-dimer, HR, Time in ER, Chlorine, eGFR, Location of the hematoma, Hematoma volume

^a^ Variables are listed according to the importance. ^b^ Because of the mechanism of SVM, the importance of variables cannot be accessed. AMC, absolute monocyte count; ANC, absolute neutrophil count; BG, blood glucose; BT, body temperature; CatBoost, Category Boosting; DM, diabetes mellitus; eGFR, estimated glomerular filtration rate; ER, emergency room; GCS, Glasgow Coma Scale; HR, heart rate; IVH, intraventricular hemorrhage; LR, logistic regression; RF, random forest; SVM; support vector machine; TG, triglyceride; WBC, white blood cell; XGBoost, extreme gradient boosting.

## Data Availability

The data presented in this study are available on request from the corresponding author. The data are not publicly available due to data-safety restrictions.

## Supplementary Materials

The following are available online at https://www.mdpi.com/article/10.3390/jpm12010112/s1. Supplementary Table S1: The comparison between the ML models and the ICH score. Supplementary Code: The intact algorithms.
