# Supplementary material for: Machine Learning-Based Approaches for Prediction of Patients’ Functional Outcome and Mortality after Spontaneous Intracerebral Hemorrhage
Source: J Pers Med. 2022 Jan 14;12(1):112. doi: 10.3390/jpm12010112 (PMC8778760; doi:10.3390/jpm12010112)
Supplement: Supplementary file 1 [file jpm-12-00112-s001.zip › Supplementary Table S1.pdf]

Supplemental Table S1. The comparison between the ML models and the ICH score. Algorithms' parameters. This table illustrates parameter details of each algorithm.

Predictive models for 90-day functional outcome

Algorithms	Parameters
Logistic Regression	{'C': 1.0, 'class_weight': 'balanced', 'dual': False, 'fit_intercept': True, 'intercept_scaling': 1, 'l1_ratio': None, 'max_iter': 100, 'multi_class': 'auto', 'n_jobs': None, 'penalty': 'l2', 'random_state': None, 'solver': 'lbfgs', 'tol': 0.0001, 'verbose': 0, 'warm_start': False}
Logistic Regression CV	{'Cs': 10, 'class_weight': 'balanced', 'cv': None, 'dual': False, 'fit_intercept': True, 'intercept_scaling': 1.0, 'l1_ratios': None, 'max_iter': 100, 'multi_class': 'auto', 'n_jobs': None, 'penalty': 'l2', 'random_state': None, 'refit': True, 'scoring': None, 'solver': 'liblinear', 'tol': 0.0001, 'verbose': 0}
Support Vector Machine	{'C': 10, 'break_ties': False, 'cache_size': 200, 'class_weight': 'balanced', 'coef0': 0.0, 'decision_function_shape': 'ovr', 'degree': 3, 'gamma': 2e-05, 'kernel': 'rbf', 'max_iter': -1, 'probability': True, 'random_state': None, 'shrinking': True, 'tol': 0.001, 'verbose': False}
Random Forest	{'bootstrap': True, 'ccp_alpha': 0.0, 'class_weight': 'balanced', 'criterion': 'gini', 'max_depth': 3, 'max_features': 'auto', 'max_leaf_nodes': None, 'max_samples': None, 'min_impurity_decrease': 0.0, 'min_impurity_split': None, 'min_samples_leaf': 30, 'min_samples_split': 2, 'min_weight_fraction_leaf': 0.0, 'n_estimators': 42, 'n_jobs': -1, 'oob_score': False, 'random_state': 0, 'verbose': 0, 'warm_start': False}
Extreme Gradient Boost	{'base_score': 0.5, 'booster': 'gbtree', 'colsample_bylevel': 1, 'colsample_bytree': 1, 'gamma': 13, 'learning_rate': 0.01, 'max_delta_step': 0, 'max_depth': 4, 'min_child_weight': 5, 'missing': None, 'n_estimators': 161, 'n_jobs': -1, 'nthread': None, 'objective': 'binary:logistic', 'random_state': 0, 'reg_alpha': 0.59,

	'reg_lambda': 1, 'scale_pos_weight': 2.792929292929293, 'seed': 2021, 'silent': True, 'subsample': 0.4}
Category Boost	{'iterations': 140, 'learning_rate': 0.05, 'depth': 3, 'l2_leaf_reg': 1, 'thread_count': 4, 'random_seed': 0, 'silent': True, 'scale_pos_weight': 0.64}

Predictive models for 90-day mortality

Algorithms	Parameters
Logistic Regression	{'C': 1.0, 'class_weight': 'balanced', 'dual': False, 'fit_intercept': True, 'intercept_scaling': 1, 'l1_ratio': None, 'max_iter': 100, 'multi_class': 'auto', 'n_jobs': None, 'penalty': 'l2', 'random_state': None, 'solver': 'lbfgs', 'tol': 0.0001, 'verbose': 0, 'warm_start': False}
Logistic Regression CV	{'Cs': 10, 'class_weight': 'balanced', 'cv': None, 'dual': False, 'fit_intercept': True, 'intercept_scaling': 1.0, 'l1_ratios': None, 'max_iter': 100, 'multi_class': 'auto', 'n_jobs': None, 'penalty': 'l2', 'random_state': None, 'refit': True, 'scoring': None, 'solver': 'lbfgs', 'tol': 0.0001, 'verbose': 0}
Support Vector Machine	{'C': 1, 'break_ties': False, 'cache_size': 200, 'class_weight': 'balanced', 'coef0': 0.0, 'decision_function_shape': 'ovr', 'degree': 3, 'gamma': 2e-05, 'kernel': 'rbf', 'max_iter': -1, 'probability': True, 'random_state': None, 'shrinking': True, 'tol': 0.001, 'verbose': False}
Random Forest	{'bootstrap': True, 'ccp_alpha': 0.0, 'class_weight': 'balanced', 'criterion': 'gini', 'max_depth': 3, 'max_features': 'auto', 'max_leaf_nodes': None, 'max_samples': None, 'min_impurity_decrease': 0.0, 'min_impurity_split': None, 'min_samples_leaf': 1, 'min_samples_split': 4, 'min_weight_fraction_leaf': 0.0, 'n_estimators': 135, 'n_jobs': -1, 'oob_score': False, 'random_state': 0, 'verbose': 0, 'warm_start': False}

Extreme Gradient Boost	{'base_score': 0.5, 'booster': 'gbtree', 'colsample_bylevel': 1, 'colsample_bytree': 0.9, 'gamma': 8, 'learning_rate': 0.01, 'max_delta_step': 0, 'max_depth': 3, 'min_child_weight': 7, 'missing': None, 'n_estimators': 226, 'n_jobs': -1, 'nthread': None, 'objective': 'binary:logistic', 'random_state': 0, 'reg_alpha': 0.7, 'reg_lambda': 1, 'scale_pos_weight': 8.881578947368421, 'seed': 2021, 'silent': True, 'subsample': 0.8}
Category Boost	{'iterations': 140, 'learning_rate': 0.05, 'depth': 5, 'l2_leaf_reg': 5, 'thread_count': 4, 'random_seed': 2021, 'silent': True, 'scale_pos_weight': 7}
